# Abundance, Fishing Season and Management Strategy for Blue Swimming Crab (*Portunus pelagicus*) in Pangkajene Kepulauan, South Sulawesi, Indonesia

**DOI:** 10.21315/tlsr2018.29.1.1

**Published:** 2018-03-02

**Authors:** Eko Sri Wiyono, Ihsan

**Affiliations:** 1 Department of Fisheries Resources Utilization, Faculty of Fisheries and Marine Science, Bogor Agriculture University, Indonesia; 2 Department of Fisheries Resources Utilization, Faculty of Fisheries and Marine Science, Indonesian Muslim University, Makassar, Indonesia

**Keywords:** abundance, blue swimming crabs, fishing season, Pangkajene Kepulauan

## Abstract

In order to manage blue swimming crabs in Pangkajene Kepulauan, management measures are required. Since the environment which affects the abundance of the blue swimming crab varies seasonally, it is necessary to take into account the seasonal nature with the aim of developing a management strategy. The objectives of this study are to define the abundance of and fishing season of blue swimming crabs in the Pangkajene Kepulauan waters, South Sulawesi, Indonesia. The fishing season was analysed using seasonal index analysis, while fish abundance was analysed by means of Equilibrium-Schaefer. The result of this study demonstrated that fishermen allocate their fishing gear all year, although the fish catch is seasonal. Based on analysis of the result, the fishing season for the blue swimming crabs is short. The peak fishing season starts in May and finishes in June. However, in order to enable their families to earn a living, fishermen operated their fishing gear throughout the year. As a result, both catch landing and effort were close to maximum sustainable yield (MSY). In order to reduce fishing pressure, it is necessary to reduce fishing gear and have a seasonal arrangement regarding fishing gear allocation.

## INTRODUCTION

The blue swimming crab (*Portunnes pelagicus*) is one of the major commodities for export with regards to the Indonesian fisheries sector. In 2012, the export value of the blue swimming crab reached USD329.724 and was the third largest fisheries export product (excluding other fish) in total value, in Indonesia (Ministry of Marine Affairs and Fisheries, Indonesia, 2012). However, research reported that the blue swimming crab faced several problems in Indonesian waters concerning stock (Kembaren *et al.* 2012; Muhsoni & Abida 2009; Wiyono & Ihsan 2015; Zairion *et al.* 2014). Increases in the type, number, size and efficiency of gears have significantly increased the fishing pressure on available stocks. Additionally, stocks of the blue swimming crab have drastically reduced and the average catch size is becoming smaller, whilst fishing grounds are becoming more distant from fishing bases. Conversely, fishermen are finding it increasingly difficult to catch blue swimming crabs. In 2010, the Indonesia Ministry of Marine Affairs and Fisheries (MMAF) reported that the number of fishermen at 2,1 million and the number of fishing boats at 588,390. Due to increasing in size and number of fishing boat, number of fisherman in 2014 increased to 2.74 million to operate 625,633 fishing boats (MMAF Indonesia 2015). MMAF also reported that 26% of fishing boats in 2014 were ‘unmotorised’, down from 30% in 2010. Conversely, fishing boat with inboard machine increased from 26% in 2010, to 30% in 2014.

A number of the warnings mentioned above indicate that stocks of the blue swimming crab in Indonesian waters have several problems concerning overfishing and an excess of effort capacity. In order to stop the decline, it is important to take action by implementing appropriate management mechanisms. Seeing as the environment can vary substantially over time, the population dynamics of fish populations are also dynamic seasonally. Growth rates, recruitment, natural mortality rates vary from season to season and affect the availability of fish resources (Cochrane 2002). Besides being characterised by great spatio-temporal variation, diversity of gears, diversity of target species and the scattering of fishing activity along coasts, small scale fisheries in tropical areas also consist of high uncertainty in relation to catch landings (van Oostenbrugge *et al.* 2002). Hence, fisheries managers require management measures to consider the impact of the seasonal variation of the population dynamics regarding fishing activity.

In this research, we studied the abundance and seasonal fishing seasons in the waters of Pangkajene Kepulauan, South Sulawesi. Statistics issued by the Indonesian Fisheries (Ministry of Marine Affairs and Fisheries 2011) reported that the waters of South Sulawesi make a significant contribution (9.3%) to national blue swimming crab production and moreover, that Pangkajene Kepulauan contributes in the region of 20% to South Sulawesi. The blue swimming crabs fisheries sector is typically small scale and operates gillnets, traps and mini trawler nets in shallow waters. In 2010, from 2317 fishing boats which operated fisherman, gillnets were dominated about 48% of total fishing gear, while traps and mini trawler nets contributed 3% and 2,8%, respectively. As respons to the annual dynamic and decreasing catch, proportion of gillnets to total fishing gears (3,767 units) were decreased (about 26%) of total fishing gear in 2014, while traps and mini trawler nets were increased 7.2% and 3.0%, respectively.

The aim of this study is to define the fishing season and the abundance of blue swimming crabs in the waters of Pangkajene Kepulauan, South Sulawesi, Indonesia. More precisely, the objective is to study 1) the annual dynamics of effort, fish catch and fish productivity; 2) the seasonal dynamics related to the seasonal pattern of fishing; and 3) stocks of blue swimming crabs. We subsequently use these results to recommend management strategies for the reduction of excessive fishing efforts.

## MATERIAL AND METHODS

### Fisheries in the Study Area

The data used in this paper are from the fisheries statistical data for Pangkajene Kepulauan from 2009–2015 and moreover, from record collector’s books from 2013–2015. The abundance of fish and annual fisheries dynamics were analysed based on fisheries statistical data, which contains reports regarding catches and effort by trips for gears annually. Prior to data analysis, the data were already separated into blue swimming crab fisheries and others. The seasonal fishing season and monthly dynamics related to the fisheries were analysed based on record collector’s books. Generally, fishermen in Pangkajene Kepulauan sell their catches to the collectors every day. Due to social relationship reasons, fishermen sell their catches to certain collectors, which provide them with an advantage.

**Figure 1:**
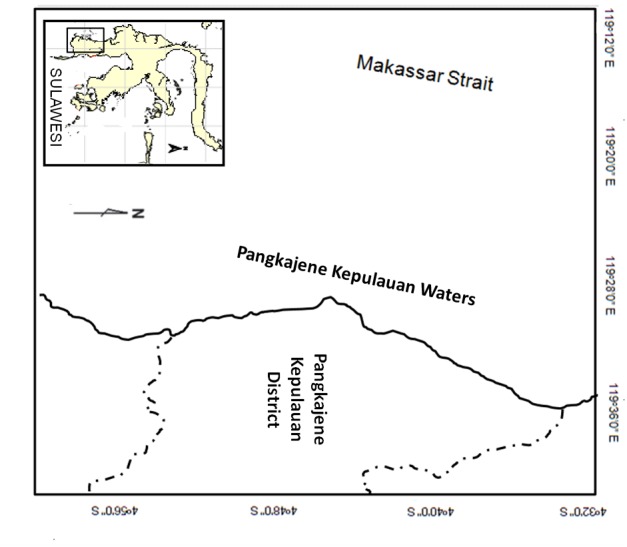
Map of Pangkajene Kepulauan, South Sulawesi Indonesia (Geospatial Information Agency of Indonesia 2014)

The fishing gear was a one day fishing trip operated around the Pangkajene Kepulauan coast (Fig. 1). The vessel’s trips and data on catches were used as the basis for the data. Data was collected from the fisherman every day, and subsequently aggregated into total catch per day. Furthermore, based on the total catch per day, data was aggregated to a monthly amount.

Beside fisheries data, the study also collected data on the climatic variables as potential external factors that may influence monthly fish abundance and fishing activities by fishermen. The data were obtained from the Pangkajene dan Kepulauan Regency Statistics Office.

### Data Analysis

The dynamics of the blue swimming crab’s abundance were analysed by catch per unit of effort (CPUE) both on a monthly and annual basis. In view of the fact that fishing gears used to capture blue swimming crabs are more than one fishing gear, we standardised each fishing gear by using the Fishing Power Index value. According to the objectives of the research, the data were aggregated on a monthly and yearly basis. Based on the monthly standardised gear operations (trips/month) and landing (kg/month) of gears, the monthly CPUE (kg/trips/month) of the gear were generated. Meanwhile, annual CPUE were generated by aggregated total catch (kg/year) and effort (trips/year) on a yearly basis.

The fishing seasons were analysed by means of the seasonal index (SI) method. Based on the monthly CPUE, the seasonal index for blue swimming crabs was computed by the decomposition moving average procedure (Makridakis *et al.* 1983).

(3) $${SI}_{m}=\frac{1}{K}\sum_{k=0}^{K-1}\frac{x_{m}+12_{k}}{T_{m}+12_{k}}\times 100\%$$

where *SI_m_* is the seasonal index for the month *m* (*m* = 1 to 12), k = {0,1,2…, K-1} K being the number of seasons for the whole time series, x*_m_*_+12_*_k_* is the raw data for the CPUE (kg/trips) in months *m*+12*k*, and T*_m_*_+12_*_k_* is the corresponding trend value estimated by centred 12 moving average procedures. By applying the maximum numbers of consecutive seasonal index (CSI), the lengths of the fishing seasons were estimated. CSI is a number which demonstrates consecutive values with regards to the seasonal index ≥ 100%. If the number is closer to 12, this indicates that the patterns related to the fishing season for species were relatively stable throughout the year; however, an index closer to 1 indicates that gears species landing were more seasonal (Ulrich & Andersen 2004).

Furthermore, using the yearly effort and CPUE data, fish stocks were estimated by applying two models (Sparre & Venema 1998):

1. The Equilibrium Schaefer model
  The mathematical equation estimation of the biological parameters with the Schaefer Equilibrium model is as follows:
  (1) $${CPUE}_{t}=a-{bf}_{t}$$
  The effort (E_t_) and catch (Y_t_) at the maximum value and the Maximum Sustainable Yield (MSY) level were estimated as follows:
  (2) $$f_{MSY}=\frac{-a}{2b}$$
  (3) $$MSY=\frac{-a^{2}}{4b}$$
2. The Fox model
  The mathematical equation estimation of the biological parameters with the Fox model is as follows:
  (4) $$\text{ln }({CPUE}_{t})=c-{df}_{t}$$
  The effort (*f_t_*) and catch (*Y_t_*) at the maximum value and the Maximum Sustainable Yield (MSY) level were estimated as follows:
  (5) $$f_{MSY}=\frac{-1}{d}$$
  (6) $$MSY=\left(\frac{-1}{d}\right)\text{exp }(c-1)$$
  where *f_t_* = effort in year *t, t* = 1,2,...,7; CPUE_t_ = yield (catch in weight) per unit of effort in year t; a = the intercept; and b = the slope. Both models conform to the assumption that CPUE declines, as effort increases, nevertheless they differ in the sense that the Schaefer model implies one effort level for which CPUE equals zero, specifically when f = −a/b, whereas in the Fox model, CPUE is greater than zero for all values related to effort.

## RESULTS

### Monthly Rainfall and Number of Rainy Days

As in other parts of Indonesia, climate in Pangkajene Kepulauan is mostly determined by rainfall that is related to monsoon winds (Table 1). Climate seasons were categorised into two categories, specifically dry season (June – October) and wet season (November–May). During dry season, the climates were marked by lowest rainfall and number of rainy days. Monthly average of rainfall and number of rainy days during dry season were 85.0 mm and 7.0 days, respectively. Conversely, during wet season were marked by highest rainfall and number of rainy days. Monthly average of rainfall during wet season was 442.9 mm and number of rainy days during wet season was 17.1 (days).

### Monthly Dynamic of Effort

**Figure 2:**
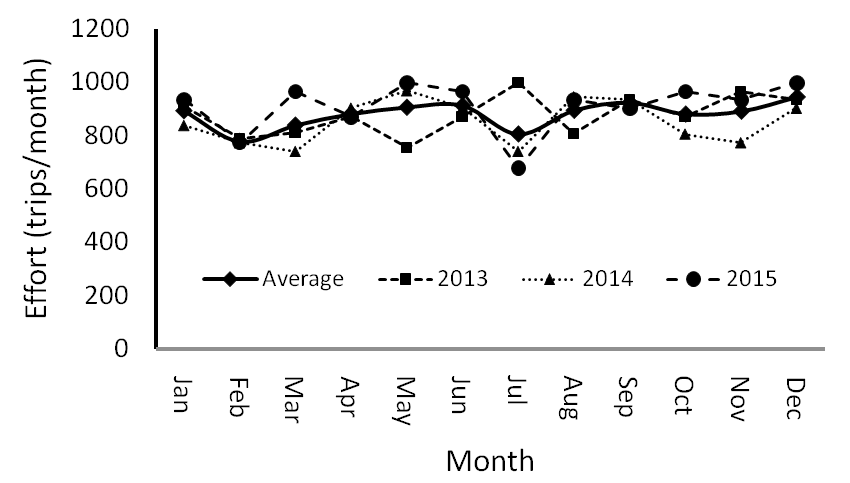
Monthly variation of effort in Pangkajene Kepulauan Monthly dynamic of catch.

Although the weather fluctuates seasonally, the average numbers of monthly trips of effort (879 ± 48.7 trips/month) are relatively stable seasonally. With regards to the fishing season, the majority of gears that capture blue swimming crabs (gillnets, mini trawls and mini trawls) were operated every day (25–28 trips/month). Although it did not have a drastic influence from March – December, except for July, the number of fishing trips (in average) tended to increase (Fig. 2). The range of fishing trips was approximately 777 trips (January) and 944 trips (December). In contrast, when comparing the years, average fishing trips illustrates an increased trend. The average trips in 2013 were in the region of 875 trips/month. This increased to 909 trips in 2015.

### Monthly Dynamic of Catch

**Figure 3:**
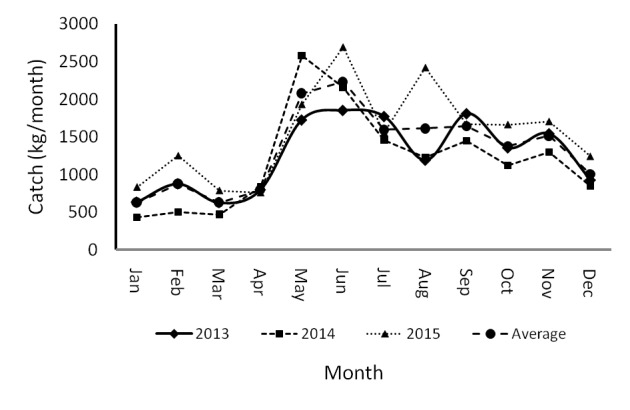
Monthly dynamics of blue swimming crabs landings.

In general, catch landings of blue swimming crabs have varied temporally in the last three years (Fig. 3). Comparing this to other years, the total catch in 2015, which ranged from 19.08kg/trips (in February) to 99.47kg/trips (in November), was the highest among gears. Meanwhile, the range of catch regarding lowest total catch (2014) varied between 435.4kg (January) – 2578.6kg (May). Comparing the years, the dynamics pattern in relation to catches reveals a similar pattern. On average, from January (634 kg) to July (2,233 kg) catches tend to increase. Then in the August (1,596 kg) – December (1.006 kg) periods the catches tend to decrease.

### Abundance

#### Monthly dynamic for CPUE

**Figure 4:**
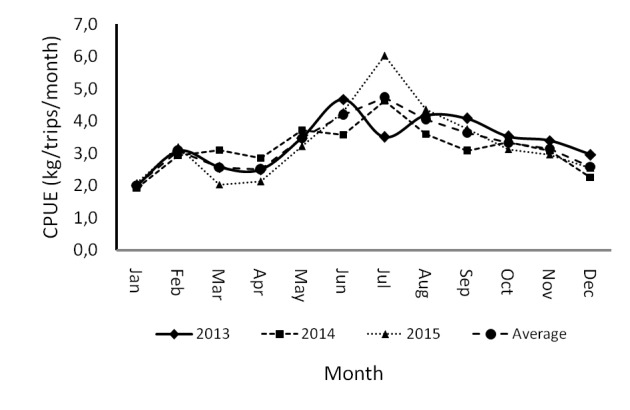
Monthly dynamics of CPUE

Three major types of fishing gear were employed to capture blue swimming crabs in Pangkajene Kepulauan, i.e., gillnets, traps and mini trawlers. Seeing as the productivity of the gillnets was highest in contrast to the other fishing gears, the fishing gears were standardised to gillnets. On average, the CPUE for blue swimming crabs in 2013 (3.33 kg/trips) was at its highest in contrast to other years (Fig. 4). During 2013, the CPUE fluctuated seasonally from 202kg/trips (January) – 4.67kg/trips (July). Meanwhile, the lowest CPUE occurred in 2014. During 2014, the CPUE fluctuated between 1.93kg/trips (January) – 4.63kg/trips (July). The average monthly CPUE for blue swimming crabs fluctuated seasonally. In total, the average monthly CPUE for blue swimming crabs was 3.27±0.79 kg/trips/month. The CPUE reached its lowest value (2.02 kg/trips) in January, whereas productivity increased and achieved its highest value (4.72 kg/trips) in July. After attaining the highest value (in July), productivity decreased until it reached its lowest value (in January).

#### Annual dynamics of CPUE

**Figure 5:**
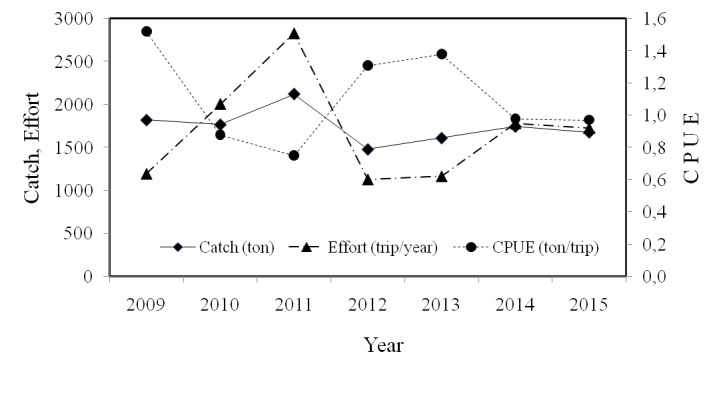
The dynamic effort, catches and CPUE in relation to blue swimming crabs in Pangkajene Kepulauan, South Sulawesi

The total fishing effort which was already standardised into gillnets showed that the allocation of fishing efforts was dynamic annually. During the 2009–2011 periods, the fishing effort increased and attained its peak number in 2011 (2826 trips/year). However, after the increasing effort did not respond with significant catches, the fishing effort tended to decrease from 2010–2015 (Fig. 5). Conversely, catches during 2009–2015 were stable and had a tendency to decrease. The increase in catches in 2011 was expected, as a response to a dramatic increase in effort, which indicated a rise in the number of catches (Fig. 5).

In view of the fact that the effort and catches were dynamic, productivity (CPUE) was also dynamic. Furthermore, from 2009–2015, productivity was dynamics although was inclined to decrease (Fig. 5). In 2009, the fishermen were able to catch roughly 1,520 ton/trips/year of fish; however, productivity decreased to 0,971 ton/trips/year in 2015.

#### Fishing Seasons

**Figure 6:**
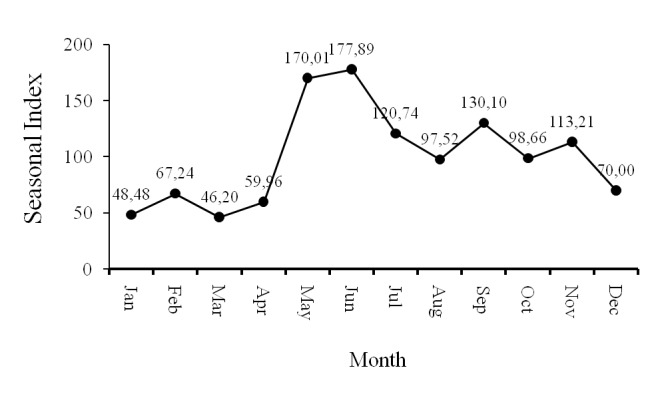
Blue swimming crab fishing season in Pangkajene Kepulauan.

The result of the seasonal index (SI) analysis confirms that the fishing season for the blue swimming crab in Pangkajene Kepulauan consists of two periods (Fig. 6). The first peak fishing season occurred between May (SI = 170.01) and July (SI = 120.74), whilst the second fishing season took place from August (SI = 97.52) to November (SI = 113.21). Thus, the blue swimming crab fishing season can be categorised as seasonal. The longest consecutive seasonal index (CSI) analysis for blue swimming crabs was 3 (May – July). This signifies that the length of the fishing season for blue swimming crabs is 3 months.

#### Maximum Sustainable Yield

Furthermore, in order to understand fish stocks, two models were selected. As a result, it was determined that the Schaefer model provides the best fit for the data. According to the Schaefer model the maximum sustainable yield (MSY) is 2,099,155 kg, whereas the effort level (f_MSY_) is 2179 standard efforts. In addition, based on the MSY and f_MSY_ values, using the precautionary principle (80% of MSY) the total allowable catch (TAC) for blue swimming crabs was 1.679.324 kg and the level of effort was 1.743 trips/year.

## DISCUSION

The result of this study demonstrated that monthly catch and abundance (CPUE) of blue swimming crab were dynamic seasonally. The catch and abundance of blue swimming crab increased from May–July and coincided with the dry season (Table 1). After reaching its peak (in June) catches were inclined to decrease, until reaching the lowest amount in December. The dynamic related to catches in tropical small scale fisheries were as expected, influenced by the weather (Wiyono *et al*. 2006; Pet-Soede *et al.* 2001). Both values for catches and abundance (CPUE), which were higher in the dry season than the rainy season indicated that catches were more abundant in the dry season. During the dry season, the mixed layers of the seas surface near the coast become shallower and caused the fishing grounds to be scattered in several locations (Wiyono *et al.* 2006). Conversely, during the rainy season, when rainfall increases, the mixed layers of the seas surface near the coast become deeper and cause both species landing and abundance to decrease.

Furthermore, the result of the seasonal index analysis indicated that the fishing season, with regards to blue swimming crabs is seasonal. The fishing season for blue swimming crabs reaches its peak from May–June (during the dry season), coinciding with peak catches and abundance. Additionally, changes to the salinity of the water and temperature drives the abundance of blue swimming crab (Kamrani *et al.* 2010) in Pangkajene Kepulauan. Juwana (2002) stated that blue swimming crabs live in salinity of 11–53 ppt. Given that the monsoon season is believed to influence water circulation seasonally, the water salinity and temperature influence and drive the abundance of blue swimming crabs (Potter *et al.* 1998). During the rainy season, when the water run-off increases, salinity in the coastal water reduces; therefore, the crabs are expected to migrate to the open ocean, which consequently causes the fishing activity of the fishermen who operate small scale fishing boats to face several challenges. Conversely, during the dry season, when the water run-off decreases, the salinity of the coastal waters becomes normal, and the crabs migrate to the coastal zone. This provides fishermen with plenty of opportunities to capture the crabs.

However, although fish catches and fish abundance were influenced seasonally, fisherman continued their fishing strategy by operating their fishing gear throughout the year (Wiyono *et al.* 2006). Less variation in fishing effort allocation during the year indicates that most fishers tended to operate their fishing gears at practically the same intensity throughout the year. It is believed that the primary reason as to why fishermen operate their fishing gear throughout the year is the responsibility to meet the needs of their families (Hilborn & Walters 1992; Pope 2002). Regarding to catch landing, depending on the fleet types, the fishermen response to the landing dynamics by different way. In short-term periods, besides reducing fishing trip during low season, the less variability of monthly fishing effort indicated that fishermen tend to change fishing ground temporally. In wet season, when fish catch reach low season, gillnet and traps fisherman waiting the good weather to operate their fishing gear by migrate to other fishing ground, while mini trawler change their fish target to capture shrimps. In long-term periods case, fisherman tend to increased their fishing boat capacity by changing their small scale fishing boat to inboard motorized medium size boats.

**Figure 7:**
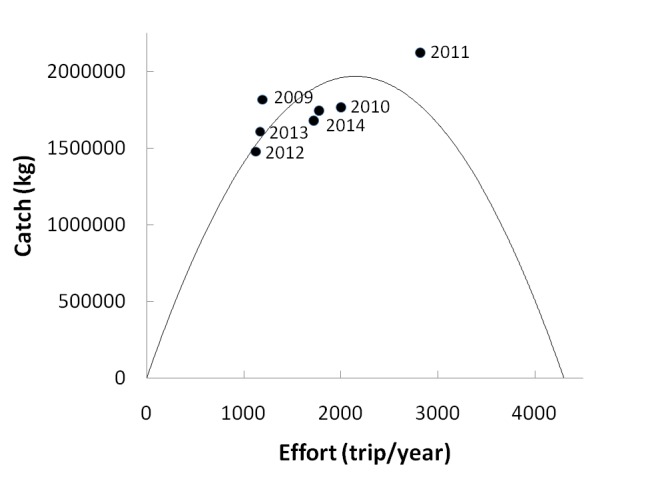
Plot catch and effort level for blue swimming crabs in Pankajene Kepulauan.

According to the Schaefer model, the result of the analysis revealed that the maximum sustainable yield (MSY) level is 2,099,155 kg/year, while the effort level (f_MSY_) is 2179 standard efforts/year. By using the precautionary principle (80% 0f MSY), the total allowable catch (TAC) for blue swimming crabs was 1,679,324 kg/year and the level of effort was 1.743 trips/year. Since the fishing pressure on blue swimming crabs has increased, catch and effort were indicated by the critical point in relation to total allowable catch (TAC). It is worth noting that over the last 7 years, fishing activity exceeded the sustainable catch on 3 separate occasions (in 2009, 2011 and 2013). This appeared to be for the reason that they want to increase the amount of catches and make more money. In 2015, the fisheries sector was in a better condition; consequently, actual catches (1,676,488 kg/year) and effort (1,726 trips/year) were under the MSY. However, if compared to TAC, the catch and effort practically exceeded the TAC value (Fig. 7).

## CONCLUSION AND MANAGEMENT STRATEGY

The result of this study demonstrated that fishermen allocate their fishing gear along year, although fish catches are seasonally dynamic, seeing as the fishing season for blue swimming crabs is very short. However, in order to continue to earn, fishermen operate their fishing gear throughout the year. As a result, both catch landing and effort are close to the maximum sustainable yield (TAC). In order to reduce fishing pressure, a reduction is required in relation to fishing gears and temporal allocation arrangements.

However, some management strategies of blue swimming crabs in the last decade was focused merely on fish biology (Kunsook *et al.* 2014; Josileen & Menon 2007; Sawusdee & Songrak 2009; Afzaal *et al.* 2016), and give little attention to the dynamic of gear. In practice, fishermen allocate or redistribute their effort (spatially or temporally) by modifying fleet, increasing technology, expanding fishing grounds to response the changing of catch and economic in fisheries (Hilborn & Walters 1992). Considering the operational needs of fisheries management, effort dynamics approach is proposed.

Given that the fishing gear was operated throughout the year, the fishing effort needs to be reduced. Fishing effort is a function of the number of fishing gear (vessel) and efficiency of the fishing gear. Therefore, the management of the fishing effort should consider both aspects. Additionally, the number of fishing gear (vessel) allocated by the fishermen depended on when and where they wanted to fish. Since the fishing effort operates all the way through the year, it should take account of seasonal fishing gear allocation throughout the year. Referring to fishing effort numbers (when fish are captured on MSY) and the seasonal index in relation to fish abundance, the monthly fishing effort allocation is proposed in (Table 2). Moreover, the fishing effort allocation fluctuates, depending on the fishing season. It is recommended that January is the minimum allocation (91 trip/month), whereas July is the maximum allocation (208 trips/month).

In contrast, fishing effort management can also be arranged by limiting the efficiency of the fishing gear. This is for the reason that the efficiency of the fishing gear will modify the fishing effort. The efficiency of fishing gear can be influenced by the size of the fishing gear, fishing tools and the ability of the fishermen. On this occasion, restrictions to the efficiency of the fishing gear can be undertaken by limiting the size of the fishing gear. For example, the number of fishing traps can be limited based on analysis of the result related to the maximum distance between fishermen’s fishing operations. Furthermore, the management objectives can also be simulated based on the fishing efficiency. If the objectives of fisheries management are to maximise the number of fishermen who work in the fisheries sector, the management alternative is to reduce efficiency, and vice versa.

In order to implement the result of management tool above, we proposed co-management as management system approach. This is because, blue swimming crabs fisheries in Pangkajene Kepulauan is characterised by small-scale fisheries that there are linkages between human and natural systems. In implementing co-management, governments cannot solve all fishery problems, but communities (fishers, government, researchers and other stakeholder) share the responsibility and authority for the management of the fishery. Community-based fisher groups are mainly pushed to participation in planning, design, implementation, monitoring and evaluation of co-management activities, while the roles of the national government and national agencies are mainly to provide technical assistance and financial assistance.

## Figures and Tables

**Table 1:** Rainfall and rainy days in Pangkajene Kepulauan.

Month	Rainfall (mm)	Number of rainy days (days)
January	940	30
February	496	21
March	444	13
April	223	14
May	119	6
June	85	7
July	-	-
August	-	-
September	-	-
October	-	-
November	199	12
December	679	24

**Table 2:** Fishing effort allocation per month.

Month	Seasonal index	Maximum allocation of fishing effort
January	63.4	91
February	93.4	134
March	77.6	111
April	75.9	109
May	106.2	152
June	121.0	174
July	144.7	208
August	119.6	172
September	109.8	157
October	106.3	153
November	100.6	144
December	81.0	116
Total		1726
